# Regulations of the key mediators in inflammation and atherosclerosis by Aspirin in human macrophages

**DOI:** 10.1186/1476-511X-9-16

**Published:** 2010-02-06

**Authors:** Li Lu, Hong Liu, Jiahe Peng, Lin Gan, Lili Shen, Qian Zhang, Liangpeng Li, Li Zhang, Chang Su, Yu Jiang

**Affiliations:** 1 Department of Biochemistry and Molecular Biology, College of Basic Medical Sciences, Third Military Medical University, Chongqing 400038, China; 2 Department of Hematology, Xin-Qiao Hospital, Third Military Medical University, Chongqing 400037, China

## Abstract

Although its role to prevent secondary cardiovascular complications has been well established, how acetyl salicylic acid (ASA, aspirin) regulates certain key molecules in the atherogenesis is still not known. Considering the role of matrix metalloproteinase-9 (MMP-9) to destabilize the atherosclerotic plaques, the roles of the scavenger receptor class BI (SR-BI) and ATP-binding cassette transporter A1 (ABCA1) to promote cholesterol efflux in the foam cells at the plaques, and the role of NF-κB in the overall inflammation related to the atherosclerosis, we addressed whether these molecules are all related to a common mechanism that may be regulated by acetyl salicylic acid. We investigated the effect of ASA to regulate the expressions and activities of these molecules in THP-1 macrophages. Our results showed that ASA inhibited MMP-9 mRNA expression, and caused the decrease in the MMP-9 activities from the cell culture supernatants. In addition, it inhibited the nuclear translocation of NF-κB p65 subunit, thus the activity of this inflammatory molecule. On the contrary, acetyl salicylic acid induced the expressions of ABCA1 and SR-BI, two molecules known to reduce the progression of atherosclerosis, at both mRNA and protein levels. It also stimulated the cholesterol efflux out of macrophages. These data suggest that acetyl salicylic acid may alleviate symptoms of atherosclerosis by two potential mechanisms: maintaining the plaque stability via inhibiting activities of inflammatory molecules MMP-9 and NF-κB, and increasing the cholesterol efflux through inducing expressions of ABCA1 and SR-BI.

## Introduction

The effects of acetyl salicylic acid (Aspirin, ASA) and other drugs in the prevention of the secondary cardiovascular complications are well known. Several studies have established guidelines for the usage of aspirin in the secondary prophylaxis of vascular diseases [1,2]. The potential roles of aspirin for primary prevention are less well understood. Meta-analysis showed that aspirin reduced the risks for both the nonfatal myocardial infarction and the fatal coronary heart disease, but did not significantly affect overall mortalities from all causes [3].

Atherosclerosis is an inflammatory disease [4] and the atherosclerotic plaques are formed by the accumulation of lipid-rich macrophages (or foam cells), cholesterols and components in the extracellular matrix (ECM). Plaque ruptures of thrombosis in the atherosclerosis are the major causes of deaths in the acute coronary syndromes and strokes [5]. The ruptures occur frequently in plaques containing soft, lipid-rich cores covered by thin and inflammatory caps of fibrous tissues [6].

There are evidences that components in the inflammatory pathway, including cyclooxygenase-2 (COX-2) [7] and its product prostaglandin E2 (PGE2), contribute to the instability of atherosclerotic plaques. MMP-9 is also related to this pathway since its expression is regulated by PGE2 in T lymphocytes and macrophage [8]. It will be also interesting to know whether other inflammatory mediators such as NF-κB play any roles in the mechanisms of atherosclerosis. In this regard, aspirin exert its function mainly by suppressing the vascular inflammation and stabilizing early atherosclerotic plaques [9], as showed earlier from the study by Cyrus's group. Further studies from the same group showed that ASA reduced the progression of atherosclerosis, and decreased the contents of cholesterols foam cells [10]. Since SR-BI and ABCA1 are the most important protein members determining the contents of foam cells, they may also be the targets of ASA.

Considering the potential connections among MMP-9, SR-BI, ABCA1 and NF-κB in related to inflammation and atherosclerosis, and the regulatory roles played by ASA in these mechanisms, we investigated the effects of ASA on the expressions of these molecules using highly relevant human macrophages.

## Materials and methods

### Materials

Phorbol ester (PMA) and acetyl salicylic acid (ASA) were obtained from Caymar (Ann Arbor, MI, USA). Tripure Isolation Reagents were purchased from Roche (German), and PCR primers were synthesized by Sagon, Shanghai. AMV Reverse Transcriptase was from Promega (Promega, Madison, USA). Scavenging Receptor BI antibody (AB396) was obtained from Cambridge Science Park (Cambridge, CB4 0FW, UK). ATP-binding cassette (ABC) transporter A1 antibody was purchased from Santa Cruz Biotechnology (Santa Cruz, CA, USA). THP-1 cells were from the China cell bank (Shanghai, China). RPMI 1640 culture medium was from Hyclone company (USA), [H^3^]-Cholesterol was obtained from GE Healthcare UK limited (Amersham Place Little Chalfont, Bucking ghamshire HP79AN UK), and the fetal bovine serum (FBS) was from Haily Biotechnology (Chengdu, China).

### Cell culture

THP-1 monocytes were cultured in RPMI 1640 culture medium with 10% fetal bovine serum (FBS). After induced by 200 nmol/L phorbol ester (PMA) for 36 hours, they differentiated into macrophages and were then maintained in RPMI 1640 medium supplemented with 5% FBS in the tissue culture incubator under 5% CO_2 _at 37°C. Cells were grown to 60-80% of the full volume of the culture flasks and then diluted with media and transferred into multiple flasks for further expansion.

### MTT assay

MTT assays were used to evaluate cell viabilities under different treatments. THP-1 macrophages in the 24-wells (Costar USA. www.scienceproducts.corning.com) culture dish were treated in 1.2 ml media with acetyl salicylic acid at different concentrations (300, 600, 1200 μM), then 20 μl of 3-(4, 5-dimethyl-thiazol-2-y)-2.5-diphenyl-tetrazolium bromide (MTT, 5 mg/ml in PBS) was added to each well. After 4 hours at 37°C, the medium was aspirated, and the formazan crystals were then dissolved by addition of 1 ml of 100 g/L SDS. Reacted media from wells were read on an ELISA plate reader at 492 nm. Since the absorbencies at 492 nm are directly proportional to the cell viabilities, so cell viabilities can be expressed as percentages of absorbencies of test cells to that of the control.

### Total RNA extraction and reverse transcription-polymerase chain reaction (RT-PCR)

Total RNAs were isolated from the control or aspirin-treated cells by the Tripure reagents (Roche, German). Total RNAs of 1 μg was reverse-transcribed to cDNAs in the RT reaction according to the manufacturer's protocol. Briefly, each RT reaction was performed in 50 μl of the reaction mix containing 5 μl of 5 × reverse transcriptase buffer with 50 mM MgCl_2_, 1 μl of 10 mM dNTP mixture, 30 U/μl RNA inhibitor, 1 μl random primers, 1 μg/μl total RNA sample. 2 μl of the cDNAs were then used for PCR amplifications. After pre-denaturation at 94°C for 10 min, the PCR reactions were carried out by 35 cycles of 94°C for 45 s, annealing at 58°C for 45 s, extension at 72°C for 45 s, followed by the final single step extension at 72°C for 10 min. The RNA of the β-actin gene was used in all reactions as internal controls for the efficiencies of the cDNA synthesis and the PCR amplification. The primer sequences used for MMP-9 were 5'-CTACCACCTCGAACTTTGACAGC-3', and 5'-CTTCCCATCCTTGAACAAATACAG-3'. The size of the PCR MMP-9 product was 494 bp. The primer sequences for the ABCA1 PCR were 5'-CCCCTGTTTCCGTTACCC-3' and 5'-AGCCCTCAGCATCTTGTC-3', and the size of the ABCA1 PCR product was 289 bp. The primer sequences for SR-BI were 5'-CTGT-GGGTGAGATCATGTGG-3' and 5'-GCCAGAAGTCAACCTTGCTC-3', and PCR product size was 216 bp. Amplified cDNA products were applied on 1.5% agarose gels, stained with Ethidium bromide and visualized under a UV light.

### Western blot analysis

THP-1 macrophages were grown in RPMI 1640 medium containing 10% fetal bovine serum and incubated with different concentrations of acetyl salicylic acid for 24 h. The cells were washed twice with phosphate-buffered saline and lysed in the protein extraction buffer (50 mM Tris-Cl, 150 mM NaCl, 1% Triton-100 with 1 mM EGTA, 2 mM EDTA and the protease inhibitors PMSF 1 mM and Atropine 5 μg/μl, and 0.1% β-ME). Total protein concentrations were determined with the Lowery method, and 30 μg total proteins were separated by 7.5% SDS-PAGE and blotted onto the nitrocellulose membrane. Blots were then incubated in 3% non-fat dry milk in PBS with 0.1% Tween-20 for 2 h to block the nonspecific binding, first antibodies were added and incubated for overnight at 4°C, followed by 2 h incubation with the respective secondary antibodies, and the specific protein bands were detected by the DAB detection. The first antibodies used were a polyclonal goat-anti-ABCA1, a polyclonal rabbit-anti-SR-BI, and a monoclonal mouse-anti-actin, respectively for each protein.

### MMP-9 activity assay by the gelatin zymography

The culture supernatants from cells treated with different concentrations of acetyl salicylic acid were mixed with 5 × PAGE loading buffer (0.5 M Tris-HCl, pH 6.8; 10% SDS, 50% glycerol and 0.5% bromophenol blue). Samples were separated in a 10% SDS-PAGE gel embedded with 5% gelatin. The gels were washed for 30 min at room temperature in a solution containing 2.5% (v/v) Triton X-100 and subsequently incubated with a reaction buffer (50 mM Tris-HCL, pH 7.5; 150 mM NaCl, 10 mM CaCl_2 _and 0.5 mM ZnCl_2_) at 37°C overnight. They were then stained for 20 min with 0.5% Coomassie brilliant blue and destained in the solution (40% distilled water, 10% acetic acid and 50% methanol) for 30 min to visualize the "clear" bands where gelatin was degraded by MMP-9 from the samples.

### Cholesterol efflux assay

HDL-mediated cholesterol efflux from THP-1 foam cells was measured as previously described [11]. THP-1 macrophages were incubated with RPMI 1640 medium containing 10% fetal bovine serum and 0.2 μCi/mL [H^3^]-cholesterol for 24 h, then became foam cells with loaded radioactive-labeled cholesterols. Various concentrations of acetyl salicylic acid (0-1200 μmol/L) were added into the fat-loaded foam cells and they were harvested 24 h later. For the equilibration of the intracellular free cholesterols, cells were washed with serum free medium, and then incubated for another 24 h with RPMI 1640 medium supplemented with 0.2% BSA. For free cholesterol efflux, cells were then incubated in serum-free medium without BSA with 50 mg/L (HDL) for up to 12 h. Then the media were collected and centrifuged before counting radioactivity. The cell monolayers were washed with PBS and lysed with water. The radioactivities from the media and the cell lysates were measured by a liquid scintillation counter. The cholesterol efflux was expressed by the H^3^-cholesterol radioactivity in the medium as the percentage of the total radioactivity from both cells and the media. Individual efflux values were averaged from three measurements in different wells.

### Densitometry and statistics

Quantifications for the relative levels of ABCA1, SR-BI, MMP-9 mRNA and protein expressions were obtained by the Quality One program. Statistical significance of the data was evaluated by Student's t test. Probability values p < 0.05 were considered significant.

## Results

**Figure 1:**
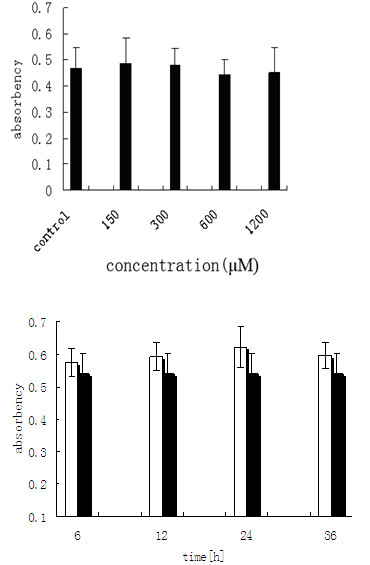
**Cell viability in the presence of acetyl salicylic acid**. Cell viabilities treated by different concentrations of ASA (A) or at different time points (B) with 600 μM ASA were determined by the MTT assays as described in Methods and Materials. Results are represented as mean±S.D from at least three experiments with duplicated samples. No changes of cell viability at different time points and concentrations of ASA.

To investigate whether acetyl salicylic acid affects the expression and activation of MMP-9, we first confirmed that ASA was not toxic to THP-1 monocytes/macrophages since it did not affect the cell viabilities within the range of concentrations tested (Figure 1).

**Figure 2:**
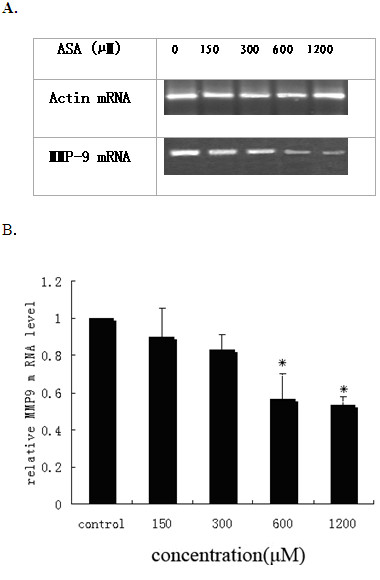
**ASA inhibition of MMP-9 expressions in THP-1 derived macrophages**. Macrophages were cultured in medium containing 10% FBS and different concentrations of ASA for 24 h. Total RNAs were isolated and mRNA levels for MMP-9 and Actin genes were examined by RT-PCR analysis. Amplified cDNA products were applied to 1.5% agarose gels, stained with ethidium bromide and visualized under a UV light (A). The signal intensities from gels were qualified by the Quantity One software and transformed to the bar graph (B). Results are expressed as mean ±S.D, of three experiments with duplicated samples. * P < 0.05,compared to control.

When the control or drug-treated cells were harvested and their mRNA levels for MMP-9 were analyzed by RT-PCR, we observed that acetyl salicylic acid inhibited MMP-9 mRNA expression in a dose-dependent manner (Figure 2). The efficiencies of the cDNA synthesis and the PCR amplification were examined by the amplification of β-actin gene fragment. We detected single bands with the predicated size (289 bp) and similar intensities for β-actin mRNAs in all RT-PCR reactions.

**Figure 3:**
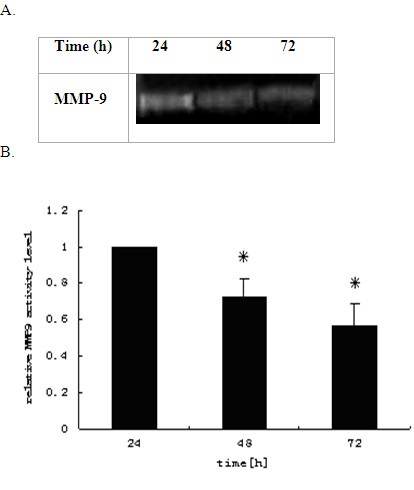
**Time-dependence of the ASA inhibition of MMP-9 activities released to media from THP-1 macrophages**. The THP-1 macrophages were cultured in media containing 10% FBS with ASA at 600 uM for 24 h, 48 h and 72 h, and the activities of MMP-9 were measured by gelatin Zymography (A). Signal intensities from the zymographs were analysed by the Quantity One software and transformed to the bar graph (B).Results are expressed as mean ±S.D, of three experiments with duplicated samples. * P < 0.05,compared to control.

**Figure 4:**
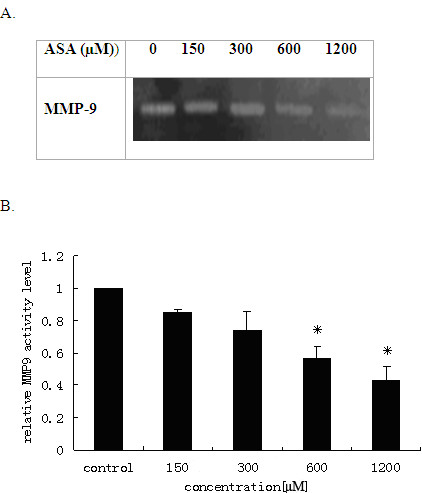
**Dose effects of the ASA inhibition of MMP-9 activities released to media from THP-1 macrophages**. The THP-1 macrophages were cultured in media containing 10% FBS with different concentrations of ASA for 24 h, and the activities of MMP-9 were measured by gelatin Zymography (A). Signal intensities from the zymographs were analysed by the Quantity One software and transformed to the bar graph (B). Results are expressed as mean ±S.D, of three experiments with duplicated samples. * P < 0.05,compared to control.

MMP-9 is an enzyme catalyzing the cleavage of cell extracellular matrix protein gelatin. We then investigated the activities of MMP-9 secreted into the culture media by the gelatin zymography. Consistent with the mRNA expression data, our results showed that acetyl salicylic acid also decreased the activities of MMP-9 secreted in the media from the treated cells both time-dependently (Figures 3) and dose-dependently (Figure 4).The activities of MMP-9 were reduced by 72% and 56% at 48 h and 72 h respectively (Figure 3B), and by 65% and 47% at 600 μM and 1200 μM respectively (Figure 4B).

**Figure 5:**
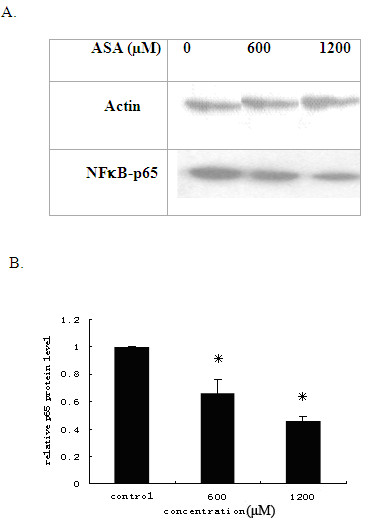
**ASA inhibition of NFκ B-p65 protein nuclear translocation**. THP-1 macrophages were treated with 0, 600, 1200 μM ASA, nuclear proteins were extracted and analysed by Western blot (A). Signal intensities in the blots were also measured by Quantity One software and transformed to the bar graph (B). The decrease at600, 1200 μM ASA was statistically significant ((P < 0.05). Results are expressed as mean ±S.D, of three experiments with duplicated samples. * P < 0.05,compared to control.

There was report that NF-κB may be involved in the atherosclerotic inflammation [12] In order to find out whether NF-κB is of the possibility that NF-κB is involved ASA decreasing MMP-9 activity or not, we also observed that ASA inhibited the NF-κB subunit p65 translocation from cytoplasm to the nucleus (Figure 5), thus inhibiting the activity of the inflammatory molecule that requires nuclear translocation for its functions. But molecular mechanism needs to study further.

**Figure 6:**
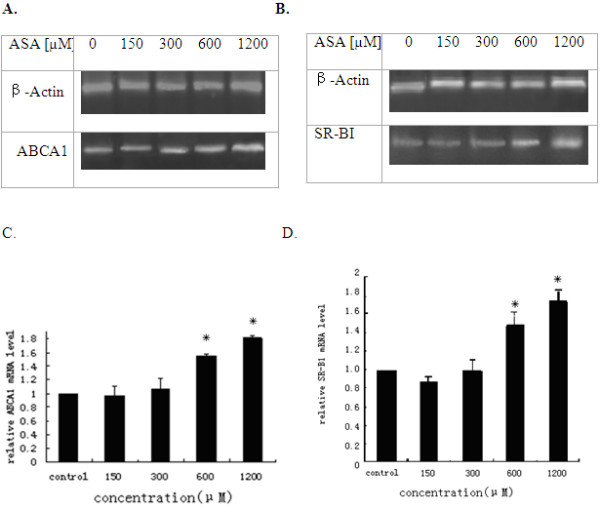
**Increases of ABCA1 and SR-BI transcripts by ASA in THP-1 derived macrophages**. THP-1 cells were cultured in media containing 10% FBS with different concentrations of ASA for 24 h. Total RNAs were isolated and mRNA levels for ABCA (A), SR-BI (B) with Actin as internal controls were examined by RT-PCR analysis. Amplified cDNA products were applied to 1.5% agarose gels, stained with ethidium bromide and visualized under a UV light (A, B). The signal intensities from gels were qualified by the Quantity One software and transformed to the bar graphs (C, D). Results are expressed as mean ±S.D, of three experiments with duplicated samples. * P < 0.05,compared to control(Figure 6C,D).

By RT-PCR, we also examined the regulations of SR-BI and ABCA1 by ASA in these same cells under similar treatment conditions. The untreated THP-1 macrophages expressed low amount of basal levels of mRNAs for both SR-BI and ABCA1 (Figure 6). Treatments with 600 μM or 1200 μM acetyl salicylic acid led to the dose-dependent increases of SR-BI, ABCA1 mRNAs. Quantification of RT-PCR data revealed that SR-BI expression increased by up to 1.74 and 1.83 fold at ASA 600 μM and 1200 μM respectively (Figure 6C), and ABCA1 expression increased by up to 1.68 and 1.83 fold at ASA 600 μM and 1200 μM respectively (Figure 6D).

**Figure 7:**
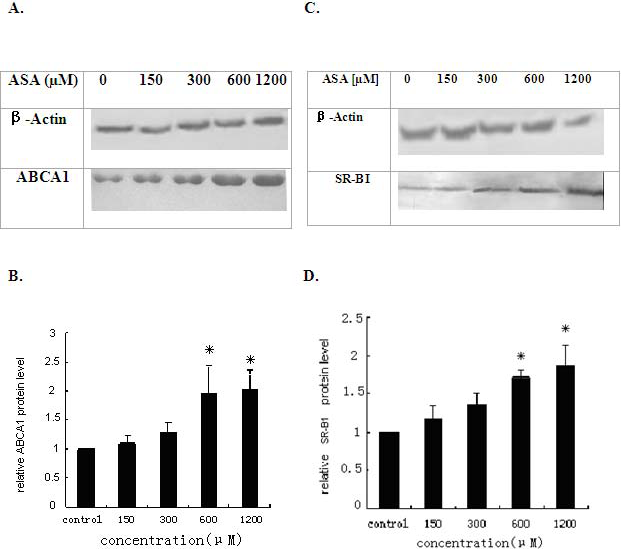
**Increases in the protein levels for ABCA1 and SR-BI from the conditioned media by ASA**. THP-1 macrophages were cultured in 1 media containing 10% FBS with ASA for 24 h. The total proteins from culture supernatants of THP-1 cells were separated by SDS-PAGE. SR-BI (A, B) and ABCA1 (C, D) protein levels were determined by Western blot analysis with specific antibodies (A, C). The signal intensities from blots were qualified by the Quantity One software and transformed to the bar graphs (B, D). Results are expressed as mean ±S.D, of three experiments with duplicated samples. * P <0.05,compared to control(Figure 7B, D).

Western blotting analysis from total lysates revealed 1.8 and 2 fold up-regulation of SR-BI protein by ASA 600 μM and 1200 μM respectively(Figure 7D), and ASA increases by up to 2.1 and 2.3 fold in ABCA1 protein at 600 μM and 1200 μM respectively(Figure 7B) from THP-1 cells.

**Figure 8:**
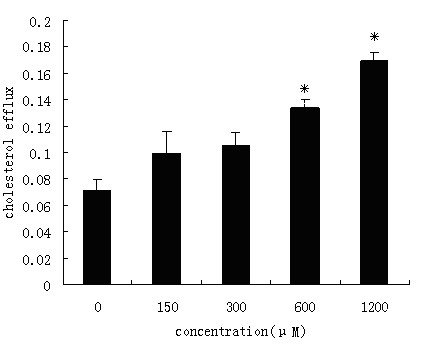
**Effect of acetyl salicylic acid on cholesterol effluxes from foam cells**. Monocyte-derived macrophages loaded with radio-labeled cholesterol were incubated for 24 h with RPMI containing 0, 150, 300, 600, 1200 μM ASA and 50 mg/L HDL. Intracellular and extracellular cholesterols were counted for their radioactivity by a scintillation counter. The decreases in the cellular contents of cholesterols caused by the ASA treatments at 600, 1200 μM were statistically significant (P < 0.05, ANOVA), whereas changes of those by ASA at 150, 300 μM are not significant (P > 0.05) between samples and the control. Results are expressed as mean ±S.D, of three experiments with duplicated samples. * P < 0.05,compared to control.

Since SR-BI and ABCA1 are also known to express in macrophages and mediate HDL-induced cholesterol efflux from these cells, we therefore tested the effect of acetyl salicylic acid on SR-BI and ABCA1 gene expressions in human monocyte-derived macrophages. In the presence of 600 μM and 1200 μM of acetyl salicylic acid, cholesterol efflux from treated cells was increased by up to 2.0 and 2.3 fold respectively, as compared to that from the untreated control cells (Figure 8).

## Discussion

Previous studies have demonstrated the potent anti-atherosclerotic and anti-metastasis activities *in vitro *and *in vivo *for the COX inhibitor aspirin [13]. However, the molecular mechanism of these actions is not clear. Recent studies indicated that MMP-9 played an important role in the development of atherosclerosis. Because MMP-9 is the principal enzyme that degrades the type IV collagen and gelatin in the extracellular matrix, it has been proposed that MMP-9 is the key factor affecting the atherosclerotic plaque stability [14]. Another recent research showed that COX inhibitors retarded progressions of established and advanced vascular atherosclerotic lesions by suppressing the formation of bioactive lipids and foam cells, so SR-BI and ABCA1, as two important mediators regulating the cholesterol contents in foam cells, may also be the targets of the COX inhibitor [10].

Our study first noted that ASA inhibited MMP-9 gene expression in THP-1 derived macrophages in the dose-dependent manner. As the important component in the atherosclerotic plaque, macrophages with lower MMP-9 expression may also secret less MMP-9 enzymes. Our work confirmed that the MMP-9 activities secreted to media from these cells were also reduced by ASA, which is consistent to the results from other researchers. Several inflammatory mediators such as IL-1, TNF-α, and ox-LDL have been shown to increase the expression of MMP-9 by macrophages [15-17], and ASA can inhibit all of these inflammatory factors. Studies showed that ASA may affect the expression of MMP-9 in cancers through the inhibition of the nuclear localizations of the AP-1 and NF-κB factors [7,18]. Our study showed that the translocation of the NF-κB subunit p65 was inhibited by ASA at 1.2 mM and NF-κB subunit p65 may be involved in the regulation of MMP-9 expression by ASA in macrophages.

In addition, Viñals group have shown that Aspirin produces an increase of CD36, ABCA1,SR-BI expressions in THP-1 macrophages by a PGE(2)-dependent mechanism [19], but we have demonstrated that the scavenger receptor class BI and ATP-binding cassette transporter A1 increased in macrophage cells in response to the ASA treatments lonely, The uptakes of modified lipoproteins by macrophages are the early steps leading to the atherosclerosis [20]. HDL-mediated reverse cholesterol transport (RCT) represents the counteracting process that may slow down the progression of foam cell formation or even induce the regression of the atherosclerosis lesions [21]. Reverse cholesterol transport involves mainly two membrane proteins: the scavenger receptor class B, type I (SR-BI) that promotes a bidirectional cholesterol movement by facilitating diffusion via its interaction with mature HDL particles [22], and the ATP-binding cassette transporter A1 (ABCA1) that promotes phospholipids and consequent cholesterol efflux by its interaction with lipid-free apoA, namely pre-HDL [23]. The integrities of both pre-HDL and the mature HDL are required for the proper functions of HDL. We found that ASA increased the expression of SR-BI and ABCA1, so reducing the cholesterol levels in foam cells and potentially slowing down the progression of atherosclerosis, which is in agreement with the observations made by Cyrus T in the low LDL receptor-deficient mice [10].

Together, our data demonstrated the important roles acetyl salicylic acid plays in the regulations of a series of key molecules involving in different aspects of inflammation and atherosclerosis. It is tempting to speculate that acetyl salicylic acid may alleviate symptoms of atherosclerosis by two potential mechanisms: maintaining the plaque stability via inhibiting activities of inflammatory molecules MMP-9 and NFκ B, and increasing the cholesterol efflux through inducing expressions of ABCA1 and SR-BI.

## Competing interests

The authors declare that they have no competing interests.

## Authors' contributions

LL and HL carried out MTT assay, RT-PCR and Western Blotting.

JP, LG carried out cell cultured.

LS, QZ, and LZ carried out MMP-9 activity assay and cholesterol efflux assay.

Lia L, CS carried out densitometry and statistics.

YJ conceived of the study, and participated in its design and coordination.

All authors read and approved the final manuscript.
